# 

**Published:** 1892-07-02

**Authors:** 


**Notices of Births, Deaths, and Marriages,** are inserted in this column at a charge of 2s. 6d. for an announcement not exceeding four lines.

### NOTICE TO CORRESPONDENTS.

All MS., Letters, Books for Review, and other matters intended for the Editor should be addressed The Editor, The Lodge, Porchester Square, London, W.

The Editor cannot undertake to return rejected M.S., even when accompanied by stamped directed envelope.

**Notice to Advertisers and Subscribers.**

All Advertisements, Orders for Copies of The Hospital, and Business Communications should be addressed to The Publisher, *not to the Editor*, The Hospital, 140, Strand, W.C.

The Cost of Subscription, post free, is as follows:—Per week, 2½d.; per quarter, 2s. 6d.; per half-year, 5s.; per annum, 8s. 6d. Foreign:—Per annum, 10s. 8d.; payable, in all cases, in advance.

**"Burdett's Hospital Annual," 1891—1892.**

Third year of publication. 600 pp. Boards, gilt lettering. A most complete guide to British and Colonial Hospitals, Dispensaries, Nursing and Convalescent Institutions, and Asylums. Price 3s. 6d. Published by The Scientific Press (Limited), 140, Strand, W.C.

**NOTICE.—The Index for Vol. XI. (ending March, 1892) is on sale at the Office of this Journal, price 2½d., post free.**

### Around the Hospitals.

### Scraps and Gleanings.

The total amount collected this year at Sleaford and district amounted to £174 16s. 1½d.

The collection of Hospital Fund at St. Mary-the-Virgin, Primrose Hill, amounted to £44 1s. 4d.

The Fishmongers' Company have given a grant of £105 to the Royal Albert Orphan Asylum, Bagshot.

Cholera has broken out in Aubervilliers, Levallois, Perret, and other manufacturing suburbs. Eight fatal cases were reported in one day.

The Grantham Hospital Saturday House-to-House Collection has resulted in the net sum of £141 11s. being handed over to the hospital, or £20 in excess of last year.

The North Staffordshire Military Band recently gave a concert in the Stubbs Walks, Newcastle, in aid of the Hospital Saturday Fund. A collection which was made at the gates realised £13 11s. 2d.

The total income of the Louth Dispensary and Hospital for the past year was £772 16s. There is still a debt of £61 0s. 10d. due to the Treasurer. During the past year 92 patients were treated in the hospital.

An exceedingly pretty bazaar, under the title of "The Old English Market Place and Fair," was opened by Lord Carrington, at the Athenæum, Camden Road, in aid of the North-West London Hospital, Kentish Town Road.

Lord Brassey presided at the annual meeting of the Sunday Society. The report recorded that art galleries and museums are now opened on Sundays in thirty-three towns in the United Kingdom and three Metropolitan districts.

On Friday and Saturday in last week two most enjoyable performances of Mrs. Neville Walford's "Queen Mab's Embassy, a Fairy Revel," were given by young people, pupils of Mrs. Wordsworth's, in aid of the Cheyne Walk Hospital for Incurable Children.

The Duchess of Portland, the Dowager Marchioness of Downshire, Lord and Lady Arthur Hill, and others, have given their patronage to a matinée which Miss Janet Steer will give in aid of the East London Hospital, on July 5th, either at Terry's or the Criterion Theatre.

On the 28th and 29th inst., the Grosvenor Club, under the patronage of the Duke of Connaught, will play the whole of Gluck's "Orféo," for the benefit of the Gordon Boys' Home. It is said that a pleasant surprise is in store for the audience in the change of scene from "Hades" to "Paradise."

Owing to the second epidemic of influenza, which was severely felt in Leicester, especially in May, the entries to the Provident Dispensary averaged 200 a week, making a total of 11,136 for the year. The Treasurer's report showed a deficiency of about £1,200, against about £1,100 last year.

The authorities of the Chelsea Hospital for Women are anxious that it should be made known to the public that the Convalescent Home at St. Leonards-on-Sea is open to patients from otherhospitals, and is for the benefit of convalescent women generally, and not only for those who have passed through the wards of the parent institution at Fulham.

The Convalescent Home, Swanley, is nearly completed. Thanks to Mr. Peter Reid, who gave £100,000, a friend of his, who gave £50,000, and other philanthropists, the cost of building the Home has not only been subscribed, but it has also been permanently endowed. The Home is intended to receive convalescents from any of the metropolitan hospitals which the trustees may select.

Sir Algernon Borthwick, presiding at the annual dinner of the West London Hospital said that its present accommodation of 101 beds ought to be increased to double that number, as many as eight persons a-day having sometimes to be turned away. £40,000 is required towards this sum. Baron Hirsch generously gave £1,000, Mr. Froy, £525; Sir Algernon Borthwick, £100; and Major General Goldsworthy, £105.

Princess Louise opened the Victoria Hospital Convalescent Home, Broadstairs, and also dedicated the Public Gardens. The Home is a pretty building in the Elizabethan style, built to accommodate 50 patients. Luncheon was provided for the Princess and suite, after which an inspection of the Home was made. Then followed the Dedication Service by the Bishop of Dover in presence of a distinguished company.

The Secretary of the City Orthopædic Hospital, Hatton Garden, appeals for funds to enable the Board of Management to add a number of additional cots for in-patients, in the adjoining site which they already possess. Many of the little incurable cripples, suffering principally from hip disease, suffer most acutely on the slightest movement of their limbs, and their pain and torture is made twofold by being carried to and from the hospital.

The Infectious Diseases Hospital for Dunfermline and West Fife is to consist of a central administrative building, flanked by two semi-detached "quarantine wards" for the reception of doubtful cases. Three isolated "fever pavilions" on the north east and west, and, lastly, a block of "offices" at the extreme western angle of the site, with a sufficient open space between them and the west fever pavilion to admit of a "future extension" in the shape of a fourth "fever pavilion" for twelve beds.

The Wellington and District Cottage Hospital, given by Mr. Egerton Burnett, was opened last week by Mr. Lawson Tait, F.R.C.S. The proceedings commenced by the donor handing the keys and deeds to Mr. Lawson Tait, who on receiving the same declared the purpose for which the meeting was assembled, and the Vicar of Wellington then offered prayer. Mr. Tait next proceeded to the front door of the hospital, and, having opened the same, declared the hospital for ever "free and open."

On Hospital Sunday hundreds of Mayfair children attended the Flower and Toy Service at Curzon Chapel, Mayfair. Cleverly concealed among the baskets of roses and other flowers were dolls and toys of every description for distribution among the metropolitan children's hospitals. The musical portion of the service was rendered by the children's orchestra, under the direction of Mr. Percy Armitage. The flowers and toys were subsequently conveyed to the children's hospitals, and many of the little contributors assisted in the distribution.

### Books Received.

Charles Griffin and Co.

"The Wife and Mother." A Medical Guide. By Albert Westland, M.D.

"A. B. C. Holiday Guide, 1892."

W. E. Smith, London.

"Calender of the University of Sydney for 1891."

H. K. Lewis.

"Medical Electricity." By Steavenson and Lewis Jones.

"The Disinfection of Scarlet Fever." By J. B. Cargeuven, M.R.C.S

"Guide to the Administration of Anæsthetics." By Henry Davis, M.R.C.S. Second Edition.

Longmans, Green, and Co.

"Nada the Lily." By H. Rider Haggard.

"The Lord of Humanity." By F. J. Grant.

Charity Organization Society.

"New York Charities Directory, 1892."

Hadden, Best, and Co.

"Hadden's Pocket Vocabulary of Medical Terms." By Henry Payne, M.D. Second Edition.

Cassell and Co.

"A Manual of Chemistry: Inorganic and Organic." For the use of Students of Medicine. Arthur P. Luff, M.D.

Macmillan and Co.

"Dietetic Value of Bread." By John Goodfellow, F.R.M.S.

James Broadley.

"The Medical Practitioner's Cash Book." By C. R. Illingworth, M.D."

Whittaker and Co.

"The Drainage of Habitable Buildings." W. L. Beardmore.

### Periodicals.

Sunday at Home, Leisure Hour, Girl's Own Paper, Boy's Own Paper, Indoor Games and Recreation, Girl's Own Indoor Book, Babyhood, The Therapist, William the Silent, The Forgotten Chest, Our Little Dots, The Child's Companion, On the Line, Light in the Home, Friendly Greetings. (Religious Tract Society). Excellent Women Series, Nos. 1 and 2. (Wilson, Hartnell, and Co., Dublin). Health Record, The Journal of Nervous and Mental Diseases, Review of Reviews, Service of the King, Child's Guardian. The Pharmaceutical Journal and Transactions. (J. A. Churchill.) James Murray's Royal Asylum, Perth, Magazine. The Pharmaceutical Journal of Australasia. The Cottager and Artisan. (Leisure Hour Office.) The Whitehall Review. (Andrew Fenwick.) The Bacteriological World and Modern Medicine. (Modern Medicine Publishing Company.) The Vaccination Enquirer. Light. The Toilers of the Deep. Electrical Review. The Edinburgh Medical Missionary Society Quarterly Paper.

### THE STUDENT OF MEDICINE.

[All communications for this Supplement should be addressed to THE EDITOR of THE HOSPITAL, at 140, Strand, with the words, "Students' Column," written in the left hand top corner of the envelope.]

### APPOINTMENTS.

Abram, George Stewart, M.R.C.S., L.R.C.P., appointed Assistant House Surgeon to the Devon and Exeter Hospital. Allardice, William C., M.B., C.M.Glas., appointed Junior House Surgeon to the Macclesfield General Infirmary. Bowlan, Marcus Marwood, M.B., B.S.Durh., L.R.C.S.Edin., appointed Assistant Medical Officer to the parish of St. George-in-the-East Infirmary. Campbell, J. Munro, M.B., C.M.Glas., appointed Medical Officer of the Newtown and Llanidloes Union Workhouse. Craig, W. Wallace, L.R.C.P.Lond., M.R.C.S.Eng., appointed House Surgeon to the Salop Infirmary, Shrewsbury. Ensor, Charles W., L.R.C.P.Lond., appointed Senior Assistant Medical Officer to the Cheshire Asylum, Macclesfield. Hubert C. Bristowe, M.D.Lond., appointed Junior Assistant Medical Officer at the Somerset and Bath Asylum, Wells. Humphry, A. D., M.R.C.S., L.R.C.P., appointed House Surgeon to the West London Hospital. Macleod, A.L., M.A., M.B., C.M.Glas., appointed Senior House Surgeon to the Macclesfield General Infirmary. Napier, A. D. Leith, M.D.Aber., M.R.C.P.Lond., F.R.S.Ed., appointed Assistant Physician to the Royal Maternity Charity. Purslow, C. E., M.D.Lond., appointed Ingleby Examiner, Queen's College, Birmingham, for the current year. Russell, J. W., M.B., B.C.Cantab., appointed Assistant Physician to the General Hospital, Birmingham. Smith, J. G., M.B., C.M., appointed Clinical Assistant, Dundee Royal Lunatic Asylum. Willey, C. H., M.D., D.Sc. (Pub. Health) Edin., M.R.C.S., appointed Honorary Medical Officer to the Sheffield Children's Hospital.

### VACANCIES.

The following vacancies are announced this week, the amount of salary in each case being given after the name of the institution:

### Resident Medical Officers.

Bristol Hospital for Sick Children and Women, St. Michael's Hill, House Surgeon, doubly qualified.

Children's Hospital, Steelhouse Lane, Birmingham, Resident Medical Officer—Salary, £80 per annum, with board, &c.

Children's Hospital, Steelehouse Lane, Birmingham, Assistant Resident Medical Officer—Salary, £40 per annum, with board, &c.

Dundee Royal Lunatic Asylum, Assistant Medical Officer—Salary, £100 per annum, with board, &c.

East London Hospital for Children, Glamis Road, Shadwell, E., House Surgeon—Board, &c.

East London Hospital for Children, Glamis Road, Shadwell, E., House Physician—Board, &c.

General Hospital, Birmingham, Assistant House Surgeon—Board, &c.

General Hospital, Birmingham, Resident Surgical Officer, doubly qualified—Salary, £130 per annum, with board, &c.

Hereford County and City Asylum, Locum Tenens, Assistant Medical Officers—Salary, £2 2s. a week, with board, &c.

Hospital for Consumption and Diseases of the Chest, Brompton, Resident House Physicians.

Royal London Ophthalmic Hospital, Moorfields, E.C., Senior House Surgeon—Salary, £75 per annum, with board, &c.

Royal South Hants Infirmary, Southampton, Assistant House Surgeon—Board, &c.

### Non-Resident Medical Officers.

Borough of Scarborough, Medical Officer of Health, not less than 25 or more than 40 years of age—Salary, £325 per annum. Will have to act also as Surgeon to the Borough Police Force, and Public Analyst, at a further salary of £25 per annum.

Dental Hospital of London, Leicester Square, W.C., Assistant Dental Surgeon; must be L.D.S.

Dental Hospital of London and London School of Dental Surgery, Leicester Square, Demonstrator—Honorarium, £50 per annum.

Hospital for Diseases of the Throat, Golden Square, W., Registrar and Pathologist—Honorarium, £25 per annum.

St. Thomas's Hospital Medical School, Obstetric Tutor—Salary, £50 per annnm.

### PASS LISTS.

### University of Cambridge.

SECOND EXAMINATION FOR MEDICAL AND SURGICAL DEGREES. EASTER TERM, 1892. PART I.

The following were examined and approved:—

PHARMACEUTICAL CHEMISTRY.—Ds. P. E. Barber, Cai.; Ds. P. F. Barton, Joh.; Ds. Blandford, Pemb.; G. F. Briggs, Joh.; Edmondson, Clare; Ds. A. E. Elliott, Joh.; Gilmore, Christ's; J. C. W. Graham, Trin.; A. E. Harrisson, Magd.; Holmes, Joh.; Lees, Cai.; Lord, Joh.; McAllum, Christ's; Muir, King's; Orton, Joh.; Rae, Joh.; Ds.

### The Hospital Advertiser.

### THE STUDENT OF MEDICINE—(*continued*).

Roberts, Down; R. J. Rowland, Corpus; Sandiland, Trinity, Scaping, Clare; Senior, Down; W. C. P. Smith, Down; Tuckett, Trin.; F. F. Ward, H. Selw.; Watson, Pet.; Wilkinson, Emman.; Ds. Worthington, Pemb.; Ds. Wrangham, Emman.

**Society of Apothecaries of London.** June, 1892.

The following candidates passed in:—

Surgery.—A. Baldwin, Edinburgh; W. R. Floyd, Cambridge and Liverpool; A. D. Ho.ard, London Hospital; E. Howgate, Yorkshire College, Le:ds; C. T. Parsons, St. Mary's Hospital; A. J. Russell, London Hospital; J. S. Tabb, Charing Cross Hospital; P. M. Thomas, Cleveland, U.S.A.; W. H. Waddington, Owen's College, Manchester; R. B. Williams, St. Thomas's Hospital; J. D. Willis, Owen's College, Manchester.

Medicine, Forensic Medicine, and Midwifery.—C. R. Batchellor, Queen's College, Birmingham; J. N. Dobie, Canbridge and St. Mary's Hospital; W. R. Floyd, Cambridge and Liverpool; S. D. Gill, Queen's College, Birmingham; C. T. Parsons, St. Mary's Hospital; W. F. Peacock, St. Mary's Hospital; J. A. T. White, St. Bartholomew's Hospital.

Medicine and Midwifery.—J. Edwards, London Hospital; A. Greenwood, London Hospital; W. Loades, Middlesex Hospital; T. E. Pallett, Westminster Hospital; S. J. Roberts, Guy's Hospital.

Forensic Medicine.—E. E. Frazer, Guy's Hospital: J. H. Hobling, St. Mary's Hospital; J. Kyffin, London Hospital; W. K. Steele, Guy's Hospital.

Midwifery.—J. C. Netherton, Dublin.

To Messrs. Floyd, Frazer, Gill, Kyffin, Parsons, Steele, and Tabb was granted the diploma of the Society entitling them to practise medicine, surgery, and midwifery.

### ATHLETICS.

### United Hospital Sports.

These sports were held last Saturday at Stamford Bridge. St. Bartholomew's, who have held the championship shield since 1885, suffered defeat on this occasion, as they only managed to win three events to the six of Guy's. The times, which ruled rather fast, were taken by Mr. P. Furnival. Details:

100 Yards Challenge Cup.—H. T. Bell, Guy's, 1; W. N. Barron, St. Bartholomew's, 2; J. W. Summerhayes, St. Mary's, 3. Won by a yard and a half; a foot between second and third. Time 10 2-5 sec.

880 Yards Challange Cup.—P. W. James, St. Bartholomew's, 1; A. H.y, St. Bartholomew's, 2; E. C. Pern, St. Thomas's, 3. Won by ten yards; double that distance divided the next two. Time 2min. 2 1-5 sec.

Putting the Shot.—M. Breton, St. George's, 33 ft. 10 in.

120 Yards Hurdle Race.—P. R. Lowe, Guy's, 1; J. Armson, Middlesex, 2; G. W. Thompson, St. Thomas's, 3. Won by two yards. Thompson fell at the last hurdle. Time, 17 sec.

220 Yards Race.—H. T. Bell, Guy's, 1; J. W. Summerhayes, St. Mary's, 2; W. N. Barron, St. Bartholomew's 3. Won by two yards; a yard and a half divided the others. Time, 22 3-5 sec.

Throwing the Hammer.—J. S. Macintosh, St. Bartholomew's, 84 ft. 8 in.

One Mile Challenge Cup (holder, H. A. Munro).—H. A. Munro, Guy's, 1; P. W. James, St. Bartholomew's, 2; A. S. Barrow, Middlesex, 3; A. E. Maturin, Guy's, 0; A. E. Walter, Middlesex, 0. The holder went to the front soon after the start, and never being approached beat his nearest opponent by a hundred yards; a much better race for second position ending in James's favour by four yards. Time, 4 min. 35 1-5 sec.

High Jump.—H. T. Bell, Guy's, 5 ft. 3¼ in.

440 Yards Challenge Cup (holder, W. G. Richards).—W. J. Summerhayes, St. Mary's, 1; W. G. Richards, St. Bartholomew's, 2; L. A. Williams, Middlesex, 3; W. H. Dudgeon, Guy's, 0; H. T. Roberts, Dental and Charing Cross, 0; E. C. Pern, St. Thomas's, 0. Won by four yards, a foot between the next two. Time, 52 2-5 sec.

Three Miles Challenge Cup (holder, H. A. Munro).—H. A. Munro, Guy's, 1; A. N. Wilde, St. Bartholomew's, 2; M. E. Tressider, Guy's, 0; C. H. Nicholson, Middlesex, 0. Munro was never pushed, and he beat Wilde, the only one besides himself who completed the course, by 250 yards. Time, 15 min. 17 1-5 sec.

440 Yards Handicap (open).—E. C. Bredin, scratch, 1; R. H. Pollard, 40 yards start, 2; W. Kent Hughes, 14, 3; A. G. Turner, 18, 0; F. H. Bailey-King, 26, 0; M. Crolyhart, 33, 0. Won by five yards. Time, 51 sec.

The prizes were afterwards distributed by Mrs. Richardson Cross.

### Inter-Hospital Cricket Cup.

Guy's Hospital v. St. Thomas's Hospital.—This match in the first round was played at Richmond, on June 10th, resulting in an easy win for Guy's by 221 runs. Score:

| Guy's. | |
|---|---|
| E. Reid, b Yearsley | 16 |
| C. J. Francis, c Arnold, b Yearsley | 19 |
| S. G. Layman, b Ashford | 1 |
| R. B. Stamford, b F. Thompson | 53 |
| E. T. Shorland, c Arnold, b Ashford | 9 |
| J. H. Bettington, c and b Thompson | 32 |
| M. P. Jones, b Thompson | 0 |
| H. T. Hicks, c F. Thompson, b Ashford | 36 |
| W. T. Hancock, b Yearsley | 28 |
| J. H. Joyce, b Arnold | 63 |
| P. R. Fitzhugh, not out | 21 |
| Extras | 26 |
| Total | 304 |

| St. Thomas's. | |
|---|---|
| G. W. Thompson, c Hicks, b Joyce | 3 |
| R. T. Storrar, c. Layman, b Bettington | 2 |
| F. C. Adair-Thompson, b Joyce | 21 |
| S. W. Maurice, run out | 6 |
| J. H. Yearsley, b Joyce | 0 |
| H. Knight, lbw, b Joyce | 8 |
| W. Ashford, b Hancock | 36 |
| H. Arnold, c Hancock, b Joyce | 0 |
| T. Marshall, c Fitzhugh, b Hancock | 4 |
| E. C. Peru, not out | 0 |
| H. T. Hardwick, absent | 0 |
| Extras | 3 |
| Total | 83 |

